# A Review of Erucic Acid Production in Brassicaceae Oilseeds: Progress and Prospects for the Genetic Engineering of High and Low-Erucic Acid Rapeseeds (*Brassica napus*)

**DOI:** 10.3389/fpls.2022.899076

**Published:** 2022-05-11

**Authors:** Pandi Wang, Xiaojuan Xiong, Xiaobo Zhang, Gang Wu, Fang Liu

**Affiliations:** ^1^Key Laboratory of Biology and Genetics Improvement of Oil Crops, Ministry of Agriculture and Rural Affairs, Oil Crops Research Institute, Chinese Academy of Agricultural Sciences, Wuhan, China; ^2^State Key Laboratory of Crop Breeding Technology Innovation and Integration, Life Science and Technology Center, China National Seed Group Co., Ltd., Wuhan, China

**Keywords:** erucic acid, plant resources, genetic engineering, industrial applications, FAE (fatty acid elongase), LPAT/LPAAT (lysophosphatidic acid acyltransferase), FAD (fatty acid desaturase)

## Abstract

Erucic acid (C22:1, ω-9, EA) is a very-long-chain monounsaturated fatty acid (FA) that is an important oleochemical product with a wide range of uses in metallurgy, machinery, rubber, the chemical industry, and other fields because of its hydrophobicity and water resistance. EA is not easily digested and absorbed in the human body, and high-EA rapeseed (HEAR) oil often contains glucosinolates. Both glucosinolates and EA are detrimental to health and can lead to disease, which has resulted in strict guidelines by regulatory bodies on maximum EA contents in oils. Increasingly, researchers have attempted to enhance the EA content in Brassicaceae oilseeds to serve industrial applications while conversely reducing the EA content to ensure food safety. For the production of both LEAR and HEAR, biotechnology is likely to play a fundamental role. Elucidating the metabolic pathways of EA can help inform the improvement of Brassicaceae oilseeds through transgenic technology. In this paper, we introduce the industrial applications of HEAR oil and health benefits of low-EA rapeseed (LEAR) oil first, following which we review the biosynthetic pathways of EA, introduce the EA resources from plants, and focus on research related to the genetic engineering of EA in Brassicaceae oilseeds. In addition, the effects of the environment on EA production are addressed, and the safe cultivation of HEAR and LEAR is discussed. This paper supports further research into improving FAs in Brassicaceae oilseeds through transgenic technologies and molecular breeding techniques, thereby advancing the commercialization of transgenic products for better application in various fields.

## Highlights

- This review presents a comprehensive and systematic evaluation of erucic acid production in Brassicaceae oilseeds, highlighting the factors that influence erucic acid production in genetically engineered *Brassica napus*.

## Introduction

**FIGURE 1:**
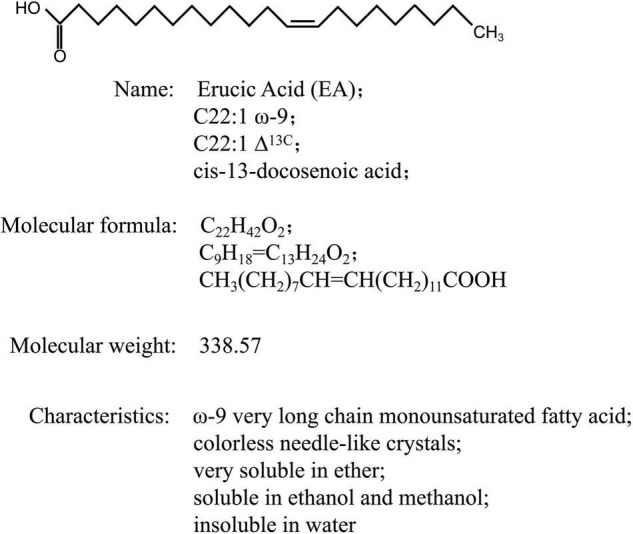
Chemical structure and other basic information of erucic acid.

Erucic acid (EA; C22:1 ω-9; C22:1 Δ13C; *cis*-13-docosenoic acid) is a very-long-chain monounsaturated fatty acid (FA) that uses sucrose, a photosynthetic product, as the main carbon source, and is formed through carbon chain lengthening and desaturation (Sakhno, 2010; Figure 1). EA is mainly present in the form of triglycerides in the fat of plant seeds. Due to its hydrophobicity and excellent lubrication properties, EA is an important oleochemical product that is widely used in various industries, and its primary use is mainly as an intermediate of fine chemicals: (1) EA can be obtained as a saturated straight-linked FA, namely behenic acid (Bährle-Rapp, 2007a), after hydrogenation reaction, and behenic acid and its derivatives can be used as plasticizers, lubricants, and stabilizers, which are widely used in the plastic industry, pharmaceutical industry, and food industry; (2) EA can be oxidized to obtain tridecanedioic acid and nonanoic acid, which are the main raw materials for synthesizing nylon-13 and nylon-1313, and can also be made into fragrance, musk, and low temperature- and light-resistant plasticizers; (3) EA derivatives also have many industrial uses, for instance, EA amide can be used as a plasticizer, anti-adhesive agent, waterproofing agent, and lubricant (Wu et al., 2007; Taylor et al., 2011); and (4) rapeseed oil with a high EA content can be used as diesel engine fuel by alkali-catalyzed transesterification and is also being used as a chemical raw material (Qi and Wang, 2009; Mcvetty and Duncan, 2015). However, in recent years, due to the promotion of double-low rapeseed (low EA and low glucosinolate), the supply of high-EA rapeseeds (HEARs) has become increasingly scarce. EA is mainly extracted from HEARs, and therefore increasing numbers of countries are focusing on HEARs and cultivating them in large quantities to meet industrial demands (Piazza and Foglia, 2001; Wu et al., 2007). Moreover, increasing the EA content in HEARs can help reduce the cost of EA production and increase its market prospects. Therefore, due to the substantial commercial value of high-EA oil in the market, further research should focus on rapeseed resources.

Both HEARs and low-EA rapeseeds (LEARs) exist, with the former being important for industry and the latter being important for health safety reasons. Before LEARs were developed, the EA content of rapeseed oil generally ranged from 45 to 50%. However, numerous zoological experiments have demonstrated that the long-term intake of large amounts of rapeseed oil with a high EA content can lead to myocardial fibrosis, cardiomyopathy, fatty deposits in the heart muscle and kidneys, stunting and retarded weight gain in animals, and can even affect male reproductive function. This is mainly attributed to the incomplete metabolism of EA in the body, resulting in the accumulation of triacylglycerols (Vles et al., 1978; Bremer and Norum, 1982; Flatmark et al., 1983; Kramer et al., 1992; Reyes et al., 2010), while heart lesions seem to be fully reversible by the avoidance of EA intake (Wallace et al., 2016). China’s national standard GB/T1536-2004 stipulates that oilseed rape with an EA content of less than 3% is considered a LEAR. In 2019, the European Commission (EU) issued regulation 2019/1870, which stipulates that the maximum content of EA in vegetable oils and fats provided to the final consumer should be no higher than 2% along with a tolerable daily intake (TDI) of 7.5 mg/kg body weight EA. Furthermore, the content of EA in camelina oil, mustard oil, and borage oil should contain no more than 5% EA, and mustard oil should contain no more than 3.5% EA (Russo et al., 2021).

Increasing numbers of studies aim to increase the EA content in Brassicaceae oilseeds to serve industrial applications. Though conventional breeding techniques to breed high-EA Brassicaceae oilseeds have increased the EA content, the maximum theoretical content of 66% has not been surpassed using conventional breeding methods (Rui et al., 2014). On the contrary, studies have also focused on reducing the EA content in Brassicaceae oilseeds to ensure food safety. Although LEAR varieties have been produced through conventional cross breeding, thereby promoting the use of many new double-low varieties (low EA and low glucosinolate), the EA content of many commercial double-low rapeseed varieties at present is generally higher than the current low-EA standard, mainly due to the mixed cultivation of rape varieties and varieties scale is more difficult to unify (Warner and Lewis, 2019).

Transgenic technology is likely to play an important role in addressing this practical issue and obtaining double-low varieties. Genetic engineering has been widely used to improve existing plant resources or produce new cultivars with desirable characteristics, thus representing a promising avenue for the breeding of HEAR and LEAR varieties (Nath et al., 2009; Huai et al., 2015; Shi et al., 2017; Qi et al., 2018; Liu et al., 2022).

In recent decades, biotechnology has developed rapidly through cutting-edge technologies such as gene editing (Zhang et al., 2020), synthetic biology (French, 2019), gene drive (Siddiqui et al., 2021), and others, resulting in many major breakthroughs and providing further technical means for genetic engineering and the realization of single-gene and multi-gene editing (Sakurai and Shindo, 2021; Nadakuduti and Enciso-Rodríguez, 2021). Further understanding the metabolic pathways of EA can provide a foundation for the quality improvement and breeding of Brassicaceae oilseeds through biotechnology.

Numerous transgenic experiments have significantly increased or decreased the EA content in recent years and these recent advances in transgenic technology are getting very close to realizing the optimal production of LEARs and HEARs. However, the available information on the engineering of EA content in Brassicaceae oilseeds is derived from isolated studies, and therefore a thorough summary and discussion of all relevant studies and reviews is required to present a comprehensive and systematic evaluation.

In this paper, we review the biosynthetic pathways of EA, introduce EA resources from plants, and summarize the available information related to the genetic engineering of EA biosynthesis in Brassicaceae oilseeds. In addition, factors affecting EA production in the genetic engineering of oilseed crops are discussed in detail, as well as the influence of the environment on EA production and the safe cultivation of HEARs and LEARs. This paper supports further work to improve the FA content of oilseed crops through transgenic technology and molecular breeding techniques, thus advancing the commercialization of transgenic products for better application in various fields.

## Erucic Acid Biosynthesis and Assembly

**FIGURE 2:**
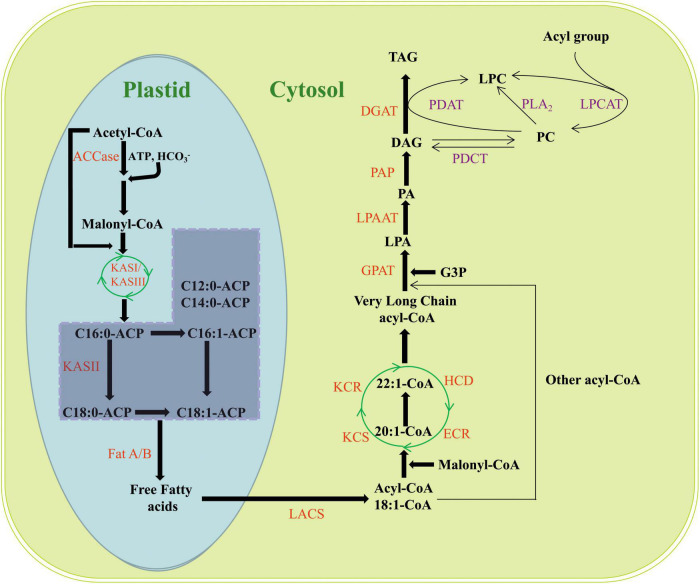
Biosynthesis and accumulation of EA in plants. ACCase, acetyl-CoA carboxylase; KAS, 3-ketoacyl-ACP synthase; LACS, long-chain acyl-CoA synthase; FAD2, fatty acid desaturase 2; FAD3, fatty acid desaturase 3; LA, linoleic acid; ALA, linolenic acid; KCS, 3-ketoacyl-CoA synthase; KCR, 3-ketoacyl-CoA reductase; HCD, 3-hydroxyacyl-CoA dehydratase; ECR, *trans*-2,3-enoyl-CoA reductase; G3P, glycerol-3-phosphate; GPAT, glycerol-3-phosphate acyltransferase; LPA, lysophospholipids; LPAAT, lysophosphatidic acid acyltransferase; PA, phosphatidic acid; PAP, phosphatidic acid phosphorylase; DAG, diacylglycerol; DGAT, diacylglycerol acyltransferase; TAG, triacylglycerol; PDAT, phospholipid diacylglycerol acyltransferase; PC, phosphatidylcholine; LPC, lysophosphatidylcholine; PDCT, phosphatidylcholine diacylglycerol cholinephosphotransferase; LPCAT, Lysophosphatidylcholine acyltransferase.

In oilseeds, FAs are *de novo* synthesized in the plastids with acetyl-coenzyme A (CoA) as the substrate, and EA is no exception. The elongation of EA starts from oleic acid (C18:1) using four core enzymes located at the endoplasmic reticulum (ER) membrane in the cytoplasm (Ohlrogge et al., 1979; Taylor et al., 2011; Li-Beisson et al., 2013; Figure 2). Finally, EA is assembled and stored as triacylglycerols (TAGs) *via* the *Kennedy* pathway (Li-Beisson et al., 2013; Chen et al., 2017; Figure 2).

Sucrose is the main carbon source for the synthesis of EA. During plant development, sucrose is converted to pyruvic acid through the Calvin cycle and further synthesized into acetyl-CoA by the pyruvate dehydrogenase complex (PDH); a precursor of FAs (Gooch, 2001). This is followed by the synthesis of malonyl-CoA catalyzed by acetyl-CoA carboxylase (ACCase), after which the malonyl group of malonyl-CoA is transferred from CoA to acyl carrier protein (ACP). Acetyl-CoA and malonyl-ACP enter the fatty acid synthesis complex (FAS) separately and undergo a sequential reaction including condensation, reduction, dehydration, and re-reduction to form C4:0-ACP, which is catalyzed by 3-ketoacyl-ACP synthase III (KAS III). Going through the same cycle reaction, the synthesis of C16:0-ACP is then catalyzed by 3-ketoacyl-ACP synthase I (KAS I) with a frequency of two carbon additions per cycle (Harwood, 2005; Li-Beisson et al., 2013). The synthesized C16:0-ACP is extended to C18:0-ACP and catalyzed by 3-ketoacyl-ACP synthase II (KAS II), following which C18:0-ACP is desaturated to form C18:1-ACP, which is catalyzed by stearoyl-ACP desaturase. C18:1-ACP is hydrolyzed and released from FAS to form free FAs by acyl-ACP thioesterases (Fat A/B). The free FAs are ultimately activated to acyl-CoA by a long-chain acyl-CoA synthetase (LACS), and then the acyl-CoA is transported to the ER and the FA chain is desaturated and extended (Salas and Ohlrogge, 2002; Bonaventure et al., 2003; Li-Beisson et al., 2013; Tjellström et al., 2013; Figure 2).

Oleic acid (C18:1) is converted to linoleic acid (C18:2) and linolenic acid (C18:3) catalyzed by fatty acid desaturase 2 (FAD2) and fatty acid desaturase 3 (FAD3), or extended to C20–C26 (including EA) very long-chain fatty acids (VLCFAs) by the FA elongation enzyme complex located at the ER membrane. The complex sequentially adds two carbon units to a growing acyl chain using four core enzymes, namely 3-ketoacyl-CoA synthase (KCS), 3-ketoacyl-CoA reductase (KCR), 3-hydroxyacyl-CoA dehydratase (HCD), and *trans*-2,3-enoyl-CoA reductase (ECR). Each elongation cycle involves four successive reactions. Malonyl-CoA and a long-chain acyl-CoA are condensed by KCS, which is then reduced by KCR to 3-hydroxyacyl-CoA, and then 3-hydroxyacyl-CoA is dehydrated by HCD and subsequently reduced to form elongated acyl-CoA catalyzed by ECR (Harwood, 2005; Haslam and Kunst, 2013; Li-Beisson et al., 2013; Huai et al., 2015; Fan et al., 2018; Figure 2).

KCS is encoded by the *FAE1* (*Fatty acid elongase 1*) gene and is a rate-limiting enzyme in the first step of the FA elongation reaction (Katavic et al., 2000); therefore, KCS is an important regulatory target for altering the EA content through genetic engineering (James et al., 1995; Qi et al., 1998; Domergue et al., 2000; Mietkiewska et al., 2004, 2007; Wang et al., 2008; Nath et al., 2009; Taylor et al., 2009; Tian et al., 2011; Shi et al., 2015, 2017; Saini et al., 2019).

After synthesis, EA is assembled and stored as TAGs, and the pathway consists of the sequential acylation and dephosphorylation of glycerol-3-phosphate (G3P). G3P is catalyzed by glycerol-3-phosphate acyltransferase (GPAT) to produce lysophospholipids (LPAs); LPA is catalyzed by lysophosphatidic acid acyltransferase (LPAT/LPAAT) to produce phosphatidic acid (PA); PA is catalyzed by phosphatidic acid phosphorylase (PAP) to produce diacylglycerol (DAG); and DAG is catalyzed by diacylglycerol acyltransferase (DGAT) to produce TAG (Stumpf and Pollard, 1983; Bates et al., 2009; Li-Beisson et al., 2013; Fan et al., 2018; Figure 2). GPAT, LPAAT, and DGAT are the three main enzymes of the *Kennedy* pathway and play very important regulatory roles in the biosynthesis of lipids and phospholipids. GPAT catalyzes the attachment of FAs on acyl-CoA to the sn-1 position of G3P, which is also the first reaction step. LPAAT catalyzes the attachment of acyl groups from acyl donors to the sn-2 position of LPA. DGAT catalyzes the formation of TAG from DAG at the sn-3 position (Taylor et al., 2011; Figure 2).

A reciprocal transformation exists between phospholipids and TAGs, which is another important pathway for TAG synthesis in plants. Phospholipid diacylglycerol acyltransferase (PDAT) allows the transfer of FAs from the sn-2 position of phosphatidylcholine (PC) to the sn-3 position of DAG, producing TAG and lysophosphatidylcholine (LPC) products (Bates et al., 2009; Lu et al., 2009; Taylor et al., 2011; Fan et al., 2018; Figure 2). PDCT mediates a symmetrical interconversion between phosphatidylcholine (PC) and DAG by catalyzing the shuffling of acyl groups between them. Through the reactions of PDCT, the acyl groups on DAG enter PC and then return to DAG after they are desaturated or otherwise modified on PC, so as to enrich PC-modified FAs in the DAG pool prior to forming TAG (Taylor et al., 2011; Hu et al., 2012; Figure 2). Lysophosphatidylcholine acyltransferase (LPCAT) catalyzes the acyl exchange at the sn-2 position of PC with the acyl-CoA pool, resulting in an enrichment of PUFA-CoAs in the acyl-CoA pool, which affords new opportunities to introduce PUFAs or other modified FAs into TAGs (Taylor et al., 2011; Figure 2).

## Erucic Acid Resources

Erucic acid is a naturally occurring long-chain FA that is found in nature mainly in the seeds of plants of the Brassicaceae and Tropaeolaceae families, but also in deep-sea fish such as trout, salmon, and cod (Ackman, 2008), which contain mainly docosenoic acid (also known as cetolic acid) accompanied by a small proportion of EA. Currently, industrial EA is mainly extracted from rapeseed oil and fish oil. Although it is possible to increase the EA content in fish liver by increasing feed intake (Lundebye et al., 2017), it is not an effective feeding strategy. For example, the feed for salmon in Norway is mainly one-third fish oil and two-thirds LEAR oil (Ytrestøyl et al., 2015), mainly because HEAR oil is often accompanied by high levels of thioglycosides, the degradation products of which are toxic and harmful. In addition, with increasing marine pollution and a growing human population, relying on aquaculture to provide EA is not a sustainable strategy (Tocher et al., 2019).

Among plants, EA is mainly found in the seeds of Brassicaceae, such as rapeseed, mustard, *Thlaspi arvense*, *Crambe abyssinica*, radish, *Lunaria annua*, *Tropaeolum majus*, and *Limnanthes alba.* Rapeseed is mainly grown in India, Canada, and Australia, and in China, it is mainly distributed in the Yangtze River Basin and southwest and northwest China. According to the agronomic traits and morphological characteristics, rapeseed can be divided into three main species (Table 1): *Brassica napus*, *B. juncea*, and *B. campestris.* Although the EA content in *B. napus* is lower than *B. juncea*, *B. napus* has strong disease resistance and high yield, while *B. juncea* is grown in drought regions and rainless mountainous areas in northwest and southwest China. *Brassica campestri*s is relatively short, its seeds fall easily, and it has weak resistance to diseases and insects and poor yield stability (Mcvetty and Duncan, 2015; Panel et al., 2016). Mustard comprises about 40 species and is one of the oldest recorded spices; it spread over thousands of years to Asia, North Africa, and Europe. Three varieties of mustard are widely used: *Brassica nigra* (black), *B. juncea* (brown), and *Sinapis alba* (white or yellow) (Vetter et al., 2020; Table 1). *Brassica carinata*, which is a hybrid of *B. nigra* and *B. oleracea*, has been planted for 6000 years mainly in western Canada and has an EA content of about 41%. However, it is characterized by a low yield, poor nutritional quality (low oleic acid, high EA, high sulfur glycosides) and long growth period (it matures 2–3 weeks later than *B. napus*) (Getinet et al., 1996; Jiang et al., 2010; Taylor et al., 2010). *Thlaspi arvense* is an annual weed in the mustard family; it has an EA content of 30–55% and prefers poor soils with some moisture in full sun, though it is tolerant of various conditions. Its seedpods shatter readily when mature (Claver et al., 2020). *Crambe abyssinica* is currently cultivated in the United States, Germany, Canada, and many other countries and has high yield potential, a short reproductive period, and a high fat and protein content. However, its cultivation area is small and thus difficult to scale, and as the seed sulfur glycoside content is high, the cake meal remaining after oil extraction cannot be used as feed (Saghai-Maroof et al., 1984; Qi et al., 2018). Radish (*Raphanus sativus*) seeds contain about 15–35% EA, and broccoli (*Brassica oleracea*) seeds contain about 50% EA. However, it is the roots or flower buds of these plants that are mainly consumed, and EA is not present in the roots or flower buds (Vetter et al., 2020). *Eruca sativa* is an annual herb with EA content of 44–46% and high level of glucosinolates; Seeds can be extracted for oil, and stems and leaves can be used as vegetables (Lazzeri et al., 2004). *Lunaria* a*nnua* is a biennial herb that grows from Europe to western Asia but is characterized by low EA yields and fragile seeds (Taylor et al., 2009; Dodos et al., 2015).

**TABLE 1:** Summary of the information including seed oil content, EA content and EA production limitations on the main plant seeds rich in EA.

Species	Oil content (%)	EA content (%)	Origin	Problems	References
*Brassica napus*	35–50	43–53	Europe	high EA content, contains glucosinolates	Mcvetty and Duncan, 2015; Panel et al., 2016
*Brassica campestris*	38–45	38–45	Asia	poor disease resistance, low yield	Mcvetty and Duncan, 2015; Panel et al., 2016
*Brassica juncea*	30–40	20–50	Europe	suitable in mountainous areas with drought and less rain	Mcvetty and Duncan, 2015; Panel et al., 2016
*Brassica nigra*	2.5–12.5	30–40	Europe	seeds are very small and mainly suitable to tropical areas	Vetter et al., 2020
*Sinapis alba*	2.5–12.5	30–40	the Mediterranean region and the Crimea	suitable in temperate climates with some humidity	Vetter et al., 2020
*Brassica carinata*	low content	30.9–45.7	Sudan in northeastern Africa and Ethiopia	low yield, poor nutritional quality, and long growth period	Getinet et al., 1996; Jiang et al., 2010; Taylor et al., 2010
*Thlaspi arvense*	28–34	30–55	Eurasia	preference for poor soils with some moisture	Claver et al., 2020
*Crambe abyssinica*	30–45	59–65	Mediterranean region	difficult to grow on a large scale, and seeds are high in sulfur glycosides	Saghai-Maroof et al., 1984; Qi et al., 2018
*Raphanus sativus*	32–52	15–35	Europe and Asia	the seeds are not consumed, only the root	Vetter et al., 2020
*Brassica oleracea*	26.3	50	southern Italy	oil has a pungent odor and the seeds are not consumed, only the flower buds	Vetter et al., 2020
*Eruca sativa*	30	44–46	South Europe and central Asia	contains a high level of thio-functionalised glucosinolates	Lazzeri et al., 2004
*Lunaria annua*	25–35	43–50	from Europe to western Asia	low yield and fragile seeds	Taylor et al., 2009; Dodos et al., 2015
*Tropaeolum majus*	6–10	75–80	South America in the Andes from Peru, Bolivia north to Colombia	difficult to obtain seeds and propagate	Taylor et al., 2009; Zasada et al., 2012
*Limnanthes alba*	20–30	12–15	Northern California, southern Oregon, and western Canada	low yields and require insect pollination to set seed	Bährle-Rapp, 2007b

In addition to Brassicaceae species, *Tropaeolum majus*, which belongs to Tropaeolaceae, is the only plant with more than 66% EA content found to date. It was introduced into Europe in the sixteenth century and elsewhere subsequently. It has an EA content of 75–80% but a low oil content of 6–10%, and seed collection and propagation are difficult (Taylor et al., 2009; Zasada et al., 2012). *Limnanthes alba* (*L. alba*), which belongs to Limnanthaceae, has a short growth habit and is adapted to growing in marshes and a cool climate. Although the seed oil EA content of *L. alba* is not high, it contains more than 95% unsaturated long-chain FAs longer than C-20, and has unsaturated bonds mainly at the Δ5 position, and therefore it has substantial antioxidant capacity. Therefore, *L. alba* seed oil is widely used in skin care and cosmetics products (Bährle-Rapp, 2007b). Through genetic manipulation techniques, it is theoretically possible to improve the EA content of *L. alba* seed oil to more than 90%.

Although there are some natural plant resources that are rich in EA, *B. napus* is the most desirable germplasm resource for meeting the industrial production of EA. The protein products from rapeseed are important sources of feed and food proteins (Nosenko et al., 2014). *B. napus* not only has strong self-compatibility and high self-fruitfulness (generally above 70–80%), as well as strong resistance to disease and leaf fall, it also has high seedling transplanting yield. It is a winter oil crop, which can be staggered with other oil crops, such as peanut and soybean. Therefore, *B. napus* has great commercial industrial value.

In addition, rapeseed oil is regarded as a nutritionally valuable edible oil on the market (Kruse et al., 2015) and has been granted Substances Generally Recognized as Safe (GRAS) status in the United States. LEAR oil occupies an important position in the food industry (Altinoz et al., 2021). LEAR oil contains a large amount of unsaturated FAs (90%), which are beneficial to human health, as well as other nutrients such as natural vitamin E. It is also stable at high temperatures, and the absorption rate of LEAR oil by the human body is up to 99%. LEAR oil is also recommended in infant formula and homemade foods for children in moderate amounts (Stimming et al., 2015; Russo et al., 2021).

More research is focusing on how to increase the EA content of *B. napus* for industrial use as well as reducing the EA content of *B. napus* for dietary use.

## Genetic Engineering of Erucic Acid Boisynthesis in Brassicaceae Oilseeds

Erucic acid is determined by the embryo genotype and is influenced by the cytoplasm (Li-Beisson et al., 2013; Liu et al., 2022). Numerous studies have shown that the inheritance of EA content is controlled by two pairs of master genes and multiple genes in cooperation with each other (Anand and Downed, 1981; Jourdren et al., 1996; Alemayehu et al., 2001), and the additive effect is significantly greater than the dominant effect. Moreover, several studies have shown that EA in rapeseeds is not only controlled by the master gene for inheritance but is also influenced by other modifier genes and the environment (Bechyne and Kondra, 1970; Wilmer et al., 1997).

Erucic acid in rapeseeds exists in the form of TAG, which can only bind to the sn-1 and sn-3 positions at the ends of the glycerol backbone and usually cannot get to the sn-2 position. Therefore, the maximum theoretical EA content is 66% (Rui et al., 2014). It is difficult to exceed this limit through conventional breeding. Therefore, the use of genetic engineering techniques to alter EA content is a pertinent research topic (Nath, 2008).

### Increasing the Extension Efficiency to Increase Erucic Acid Content

Currently, the *FAE1* gene has been cloned from *Crambe abyssinica*, *Tropaeolum majus*, *B. napus*, and *Arabidopsis thaliana* (Rossak et al., 2001; Wang et al., 2008). By cloning this gene and overexpressing it in plants, the EA content in transgenic crops can be increased to varying degrees (Table 2). The *FAE1* gene has seed-specific expression properties. In HEARs, there are two functional copies of *FAE1*, located on chromosomes A8 and C3, with more than 98% sequence similarity. The FA elongase complex is present in HEARs, whereas the activity of this complex is not detectable in LEARs, which is mainly associated with the absence of KCS enzyme activity (Roscoe et al., 2001; Puyaubert et al., 2005).

**TABLE 2:** Comparison of research on improving EA content by means of genetic engineering in Brassicaceae oilseeds.

Gene	Donor species	EA content of donor species/%	Receptor species	Expression	EA content of the control (%)	EA content of transgenic receptor species (%)	Fold-change	References
*FAE*	*Tropaeolum majus*	70–75	*Arabidopsis thaliana*	35s:*FAE*	2.1	3.2–4.0	1.7-fold increase	Mietkiewska, 2004
			*Arabidopsis thaliana*	Napin:*FAE*	2.1	9.6	4.5-fold increase	
			*Arabidopsis thaliana*		1.8 ± 0.1	11.3 ± 2.6	Sevenfold increase	
*FAE*	*Crambe abyssinica*	55–60	*Arabidopsis thaliana* (a *fae1* mutant line)	Napin:*CrFAE*	0.0 ± 0.0	10.1 ± 2.7	12-fold increase	Mietkiewska et al., 2007
			*Brassica carinata*		35.5	47.4	1.3-fold increase	
*FAE1*	*Thlaspi arvense*	38–40	*Arabidopsis thaliana*	OLE2:*TaFAE1*	< 2.5	7.43–8.56	3∼4-fold increase	Claver et al., 2020
*LPAAT*	*Limnanthes alba*	12–15	*Brassica napus*	Napin:*LPAAT*	37.7–39	37.5–41	No change	Lassner et al., 1995
*FAE*	*Arabidopsis*	1.8	*Brassica napus*	*FAE1* + *LPAAT*	43	48–53	low increase	Katavic et al., 2001
*LPAAT*	yeast	0						
*FAE1*	HEAR	52	HEAR	*BnFAE1* + *LdLPAAT*	54	63	1.2-fold increase	Nath et al., 2009
*LPAAT*	*Limnanthes douglasii*	12–15						
*FAE*	*Crambe abyssinica*	55–60	*Brassica carinata*	*CrFAE* + *FAD2*-ihpRNA	40	66.5	1.7-fold increase	Mietkiewska et al., 2008
*FAD2*	*Brassica carinata*	40						
*FAE1*	*Brassica napus*	40	*Crambe* *abyssinica*	*BnFAE1* + *LdLPAAT* + *CaFAD2*-RNAi	60	72.9–76.9	1.2∼1.3-fold increase	Li et al., 2012
*LPAAT*	*Limnanthes douglasii*	0						
*FAD2*	*Crambe abyssinica*	60						
*FAD2*	*Brassica carinata*	12	*Brassica carinata*	*FAD2-*co-supression	12	27	2.25-fold increase	Jadhav et al., 2005
	*Brassica carinata*	5	*Brassica carinata*	*FAD2-*antisense	5	19	3.8-fold increase	
*FAD2*	*Brassica napus*	42.25	*Brassica napus*	*BnFAD2*-RNAi	42.25	45.62	1.1-fold increase	Shi et al., 2017
*FAD2*	HEAR	47.26	HEAR	*BnFAD2-*amiRNA	47.26	50.37–52.38	1.1–1.8	Wang et al., 2019
*FAD2*	LEAR	0.53	LEAR		0.53	0.69–0.98	1.3∼1.8-fold increase	
*FAD2*	*Thlaspi arvense*	35	*Thlaspi arvense*	*TaFAD2-*CRISPR/Cas9	35	40	1.1-fold increase	Jarvis et al., 2021
*FAE*	*Brassica napus*	40	*Crambe abyssinica*	*CaLPAT2*-RNAi	62.5	63.1–66.3	1.0∼1.1-fold increase	Qi et al., 2018
*LPAT*	*Limnanthes douglasii*	52		*BnFAE* + *LdLPAT*		< 66.4	1.1-fold increase	
*FAD2*	*Crambe abyssinica*	62.5		*BnFAE* + *LdLPAT* + *CaFAD2*-RNAi		≤ 79.2	1.3-fold increase	
*LPAT2*				*BnFAE* + *LdLPAT* + *CaFAD2*-RNAi + *CaLPAT2*-RNAi		≤ 71.6	1.1-fold increase	

Studies have shown that different species have different types and numbers of *KCS* genes encoding different KCSs with different substrate specificities, and therefore, the EA content in different crops varies greatly (Table 2). Transfer of the *FAE1* gene from *C. abyssinica* into a zero-EA *A. thaliana* mutant increased the EA content from 0 to 12.8%. Transfer of the *FAE1* gene from *C. abyssinica* into *B. carinata* increased the EA content from 35.5 to 51.9%, while the transfer of this gene from *Tropaeolum majus* to a zero-EA *A. thaliana* mutant increased the EA content from 2.1 to 9.6%, leading to a fivefold increase.

The current selection for LEARs is based on loss-of-function mutations in *FAE1* and *FAE2* (Das et al., 2002; Wu et al., 2008; Table 3). The world’s first zero-EA rapeseed “Oro” was derived from a dramatic decrease in EA content after targeted mutation of the 845th base of *FAE1*. Insertion of the endogenous long-terminal repeat (LTR) retrotransposon BRACOPIA into the 5’ coding region of *FAE1* also led to the discovery of the world’s first low-EA *B. rapa* (Fukai et al., 2019). The EA content of *B. napus* can be reduced from 40% to less than 3% by inhibiting *FAE1* gene expression by RNA interference (RNAi). Similarly, the EA content of *B. napus* can be reduced from 40 to 0.36% by inhibiting *FAE1* gene expression by intron-spliced hairpin RNA (ihpRNA), while the EA content of *B. napus* can be reduced from 42.25 to 2.02% in HEAR and from 0.87% to undetectable levels in LEAR by inhibiting *BnaFAE1* and *BnaFAD2* (*Brassica napus fatty acid*Δ*12-desaturase 2*) expression by RNAi. In addition, the EA content was reduced to nearly zero in *B. napus* when CRISPR/Cas9 technology was used to create targeted mutations on *BnaFAE1* (*BnaA08.FAE1* and *BnaC03.FAE1*) (Liu et al., 2022; Table 3). Therefore, inhibiting the expression of *FAE* to significantly reduce the amount of EA constitutes an effective strategy.

**TABLE 3:** Comparison of research on decreasing EA content by means of genetic engineering in Brassicaceae oilseeds.

Gene	Species	EA content of species (%)	Expression	EA content of transgenic receptor species (%)	References
*FAE1.1*	*Brassica napus*	40	*BnFAE1.1-*ihpRNA	0.36	Tian et al., 2011
*FAE1*	*Brassica napus*	40	*BnFAE1-*RNAi	< 3	Shi et al., 2015
*FAE1*	*Brassica napus*	42.25	*BnFAE1*-RNAi	1.1	Shi et al., 2017
*FAD2*			*BnFAD2*-RNAi + *BnFAE1*-RNAi	2.02	
*FAE1*	*Thlaspi arvense*	35	*TaFAE1-*CRISPR/Cas9 + *TaFAD2-*CRISPR/Cas9	nearly zero	Jarvis et al., 2021
*FAD2*					
*FAE1*	*Brassica napus*	31.05–34.95	*BnaA08.FAE1-*CRISPR/Cas9 + *BnaC03.FAE1-*CRISPR/Cas9	zero	Liu et al., 2022

KCS activity was restored and EA content was greatly increased after transfer of the *FAE* gene into *B. napus* (Lassner and Lardizabal, 1996). Since KCS has different substrate specificities, to increase EA content, genes with high KCS activity should be selected.

Although the EA content of rapeseed could be increased to a certain extent using this approach, the limit of 66% cannot be exceeded because the sn-2 position of TAG cannot not bind EA.

### Increasing Assembly Efficiency to Increase Erucic Acid Content

The pathway to exceed the EA content limit of 66% is the entry of EA into the sn-2 position of TAG. LPAAT has a strong ability to transfer EA to the sn-2 position of TAG (Nath et al., 2009; Li-Beisson et al., 2013). When the *LaLPAAT* and *LdLPAAT* genes, which were cloned in *Limnanthes* spp., were transferred into rapeseed, the EA content increased at the sn-2 position and formed triglycerides, but the total EA content did not increase (Lassner et al., 1995; Table 2). This indicates that the increase of EA at sn-2 position is compensated by the decrease of EA content at the sn-1 and sn-3 positions. This result suggests that, in the absence of an increase in EA synthesis, the introduction of the *LPAAT* gene only caused a redistribution of EA at the three hydroxyl positions of glycerol (Lassner et al., 1995; Brough et al., 1996).

When *FAE* and *LPAAT* were simultaneously introduced into *B. napus*, overexpression of *FAE* and *LPAAT* ensured the insertion of EA at the sn-2 position of the glycerol backbone, resulting in only a small and non-significant increase in EA content (Katavic et al., 2001; Nath et al., 2009; Table 2). This result may be due to the presence of competitive desaturation and irreversible binding to the storage lipids, or a lack of available fatty acyl groups during FA chain extension. When *FAE* and *LPAAT* were simultaneously introduced into *B. napus* with a high EA content, the EA content increased significantly from 54 to 63%, and the recombinant F2 plants exhibited an EA content of up to 72% (Nath et al., 2009; Table 2).

Although the EA content of rapeseed can be increased and exceed the limit of 66% using this approach, industrial applications would require increases of at least 80% and above in order for EA production to be financially viable, as EA contents above 90% would greatly reduce the cost of purification (Li et al., 2012).

### Inhibiting Competing Substrates to Increase Erucic Acid Content

The overexpression of *KCS* and *LPAAT* did not significantly increase the EA content in some transgenic receptors (Table 2), likely because of the competition between FA elongation and the desaturation reaction of the same substrate, namely oleic acid. One of the metabolic pathways of oleic acid is the synthesis of EA by carbon chain lengthening under the action of the FAE1 enzyme, and the other is the synthesis of polyunsaturated fatty acids (PUFAs) such as C18:2 and C18:3 by desaturation under the action of the FAD2 enzyme, which is the first and key step in the synthesis of PUFAs. Therefore, inhibiting the expression of the *FAD2* gene is also an important way in which the EA content can be increased. Inhibition of the *FAD2* gene increases the content of oleic acid, which in turn provides sufficient substrate for EA synthesis and increases EA content in small amounts (1.1∼3.8-fold increase) (Table 2). In *B. carinata*, following the expression of the co-suppressed *FAD2* gene, the EA content was significantly increased from 12 to 27%; after antisense *FAD2* gene expression, the EA content was also significantly increased from 5 to 19% (Jadhav et al., 2005; Table 2). The EA content increased from 47.26 to 52.38% after the silencing of the *FAD2* gene by artificial miRNA in *B. napus* with a high EA content, and the EA content was increased from 0.53 to 0.98% after the silencing of the *FAD2* gene by artificial miRNA in *B. napus* with a low EA content (Wang et al., 2019; Table 2). In *B. napus*, inhibition of the *FAD2* gene by RNAi resulted in EA content increases of 42.25–45.62% (Shi et al., 2017; Table 2). In addition, the EA content increased from 35 to 40% in *Thlaspi arvense* when CRISPR/Cas9 technology was used to create targeted mutations on *TaFAD2* (Jarvis et al., 2021; Table 2).

Erucic acid is not only genetically controlled by the master gene, but is also influenced by other modifier genes. Infiltration of the multigene expression vector: *BnFAE* (*Brassica napus FAE*) + LdLPAAT (*Limnanthes douglasii LdLPAAT*) + *CaFAD2*-RNAi (*Crambe abyssinica FAD2*) into *C. abyssinica* increased the EA content to 73% in the transgenic progeny, and the individual EA content was found to be as high as 76.9% after single-seed analysis (Li et al., 2012). Therefore, genetic engineering by overexpressing *FAE* and *LPAAT*, along with the inhibition of *FAD2* gene expression, can effectively increase EA content.

### Suppressing the Expression of Endogenous *LPAT* to Increase Erucic Acid Content

The LPAT2 enzyme in Brassicaceae cannot use EA as a substrate to catalyze the incorporation of FA into triglycerides on the sn-2 position (Kuo and Gardner, 2002). To maximize the EA content of high-EA Brassicaceae oilseeds, genetic modification strategies have been developed by incorporating EA on the sn-2 position by introducing *LdLPAT* (*Limnanthes douglasii LPAT*), which can use EA as a substrate (Lassner et al., 1995). The transfer of endogenous *CaLPAT2* in *C. abyssinica* allowed an increased carbon flux to EA and less to PUFA (Qi et al., 2018). Compared with the wild-type, the EA content of the *CaLPAT2*-RNAi transgenic T1 seed oil was higher by 64.5% on average and ranged from 63.1 to 66.3% (Table 2). The infiltration of the multigene expression vectors *BnFAE* + *LdLPAT* + *CaFAD2*-RNAi and *BnFAE* + *LdLPAT* + *CaFAD2*-RNAi + *CaLPAT2*-RNAi into *C. abyssinica* resulted in EA contents of as much as 79.2 and 71.6%, respectively, in the transgenic progeny. The four-gene transformants of *BnFAE* + *LdLPAT* + *CaFAD2*-RNAi + *CaLPAT2*-RNAi presented greater carbon resource deposition into the C22:1 and C18:1 moieties and lower PUFAs when compared to the wild-type and the transformants of other vectors. This demonstrates that the suppression of endogenous *LPAT2* is a new and promising strategy for altering the EA content of Brassicaceae oilseeds (Table 2).

### Increasing Assembly Efficiency to Increase Erucic Acid Content

The expression of *DGAT* also plays an important role in the accumulation of FAs (Maisonneuve et al., 2010; Maraschin et al., 2018). DGAT, as a rate-limiting enzyme, is the catalyst of the final step of TAG biosynthesis and exclusively uses acetyl-CoA as the acyl donor. DGAT1 and DGAT2 are the main contributors to the acylation of diacylglycerols and are present in oil crops. Four isomers of each type exist, and it is therefore important to select the appropriate DGAT isomer that can contribute to the FA composition of the enzyme and enhance specific FAs. The overexpression of ricinoleic acid hydroxylase in *A. thaliana* resulted in a decrease in ricinoleic acid and oil content, whereas the coexpression of DGAT2 of ricin restored the oil content close to the wild-type levels (Burgal et al., 2008). Overexpression of *AtDGAT1* in *Jatropha* resulted in a significant increase in oil in the seeds and leaves (Maravi et al., 2016). During seed development in LEARs and HEARs, BnDGAT2 isomers were found to exhibit significant differences toward 22:1-CoA, being more active in HEARs, and BnDGAT2 produced 6–14 times more TAG using 22:1-CoA than in LEARs (Kamil et al., 2019). Most likely, these shifts are due to the selection pressure for increased oil content within breeding programs. Therefore, to increase the EA content or oil content, it is essential that DGAT specificities are optimized.

### Selection of Unique Promoters to Increase Erucic Acid Content

In addition to using the above genes to alter the EA content, the EA content can also be increased through the use of unique promoters. It is well known that the transcription level of genes is influenced not only by the gene characteristics, but may also be restricted by the promoter (Saini et al., 2019). In transgenic Brassicaceae oilseeds, the overexpression of *FAE* using powerful seed-specific promoters significantly increased the EA content in the seeds and reduced the potential risk of constitutive expression of the *FAE* gene. For example, the EA content increased from 2.1 to 9.6% when *FAE* was expressed by napin promoters in *A. thaliana*, while the EA content increased from 2.1% to 3.2%–4.0% when *FAE* was expressed by the 35S promoter in *A. thaliana* (Table 2).

## Effects of the Environment on Erucic Acid Production

In addition to genetic control, EA production is also influenced by environmental factors (Shi et al., 2003). The sowing time, climate of the planting site, lodging angle, planting density, and fertilizer all affect EA production (Yaniv et al., 1994; Uğur et al., 2010; Sanyal and Linder, 2013; Liu et al., 2015; Khan et al., 2018; Davoudi et al., 2019). The EA content of *Eruca sativa* with autumn sowing was 54.79%, while spring sowing decreased the content to 46.64% (Uğur et al., 2010). The EA content of the winter rapeseed cultivar Huayouza 62 increased from 1.13 to 1.49% when the plant lodging angle was manually increased from 0° (vertical) to 90° (horizontal) (Khan et al., 2018). The EA content was reduced from 1.46 to 1.05% when the planting density increased from 15 plants m^–2^ to 45 plants m^–2^ (Khan et al., 2018). However, the EA content did not vary significantly with different nitrogen rates (180 kg N ha^–1^ and 360 kg N ha^–1^) (Khan et al., 2018) and nitrogen forms {manure; nitrate [Ca(NO3)2, 15.5% N]; ammonium [(NH4)2SO4, 21% N]} (Uğur et al., 2010). The EA content of a LEAR differed significantly when irrigation was conducted at three levels, including routine irrigation (control), irrigation interruption at the pod formation stage, and irrigation interruption at the flowering stage. Under the three levels of irrigation, the EA contents of the Dalgan cultivar were 0.17, 0.27, and 0.41%, respectively, and the EA contents of the Hyola 401 cultivar were 0.22, 0.29, and 0.35%, respectively (Farda et al., 2018). In addition, selenium also has an effect on EA content, and after spraying rapeseed leaves with sodium selenate, the EA content of a LEAR was significantly reduced from 0.32 to 0.29% (Davoudi et al., 2019).

## Safe Cultivation of High-EA Rapeseeds and Low-EA Rapeseeds

In recent decades, oil crops and transgenic oil crops have been promoted and planted on a large scale due to improved breeding, market incentives, and interest in improving the nutritional quality of oil crops (Kramer et al., 1983; Abbadi and Leckband, 2011). Genetically modified organisms (GMOs) have been a major concern for the public and regulatory agencies. Although some opponents fear that GMOs pose risks to the ecosystem, scientists generally agree that genetically modified foods do not pose a particular risk to human consumption, and even if certain risks exist, such as allergenicity and toxicity, they have long been documented and measures have been taken to avoid such risks (Chen and Gao, 2014).

Although the price of HEARs has increased, producers are concerned that if HEARs are planted in large quantities, they will contaminate the LEARs and make it impossible to maintain low EA levels (Warner and Lewis, 2019). There are two main causes of contamination; one is the contamination of autochthonous seedlings due to crop rotation, and the other is increased EA contents through accidental pollination when HEARs are planted nearby. Unacceptable EA levels have some health and industrial impacts when transmitted through the food chain. Therefore, to avoid contamination, it is firstly very important to choose a crop rotation duration of at least 3 years, because simulations have found that it takes 16 years after harvest of a GM crop to reduce viable seeds to less than 1%. Secondly, it is important to maintain a safe distance between crops or establish strict isolation zones to minimize the effects of cross-pollination. In addition, measures are needed to manage weeds such as wild mustard (*Brassica kaber*, 31.5% EA) and wild radish (*Raphanus raphanistrum* subsp. *raphanistrum*, 26.7% EA) (Leaper and Melloul, 2011).

## Conclusion and Perspectives

Brassicaceae oilseeds not only provide common edible oils for human consumption, but also play an important role in human nutrition, alternative energy sources, and industry. Improving the nutritional value of crop products through plant genetic engineering has shown great potential and has created enormous economic and social benefits (Huai et al., 2015; Liu et al., 2021). The genetic regulation of EA content is of great importance in obtaining HEARs and LEARs. Thus far, transgenic HEARs with EA contents up to 72% are presently available and can be further developed *via* transgenic engineering to obtain higher EA content cultivars (Nath et al., 2009). LEAR oil contains a large amount of unsaturated FAs (90%), which are beneficial to human health, and thus LEAR oil is one of the most common vegetable edible oils on the market and is also recommended for infant and child nutrition due to its good FA composition (Hilbig et al., 2012; Stimming et al., 2015). Biotechnology can be used to more precisely regulate the EA synthesis pathway so as to obtain varieties with the target EA contents. This will also be of great significance to the oil crop industry and oil processing industry.

## Author Contributions

PW and FL designed and structured the review, collected the information, organized the tables, and wrote and revised the manuscript. XX prepared the figures. FL, GW, and XZ commented on the manuscript. All authors read and approved the final manuscript.

## Conflict of Interest

XZ was employed by China National Seed Group Co., Ltd. The remaining authors declare that the research was conducted in the absence of any commercial or financial relationships that could be construed as a potential conflict of interest.

## Publisher’s Note

All claims expressed in this article are solely those of the authors and do not necessarily represent those of their affiliated organizations, or those of the publisher, the editors and the reviewers. Any product that may be evaluated in this article, or claim that may be made by its manufacturer, is not guaranteed or endorsed by the publisher.
